# Development and Validation of an Optical Sensor-Based Automated Urine Flow Meter for Real-Time Patient Monitoring

**DOI:** 10.3390/s26030849

**Published:** 2026-01-28

**Authors:** Piyush Hota, Adithya Shyamala Pandian, Rodrigo E. Domínguez, Manni Mo, Bo Fu, Sandra Miranda, Pinar Cay-Durgun, Dheeraj Sirganagari, Michael Serhan, Peter Serhan, Kevin Abi Karam, Naomi M. Gades, Peter Wiktor, Leslie Thomas, Mary Laura Lind, Erica Forzani

**Affiliations:** 1 School for Engineering of Matter, Transport & Energy, Ira A. Fulton School of Engineering, Tempe, AZ 85287, USA; phota@asu.edu; 2 Department of Nephrology and Hypertension, Mayo Clinic Arizona, 5777 E Mayo Boulevard, Phoenix, AZ 85054, USA; ashyamal@asu.edu (A.S.P.); pcay1@asu.edu (P.C.-D.);; 3 Center for Bioelectronics and Biosensors, Biodesign Institute, Arizona State University, 1001 S McAllister Ave., Tempe, AZ 85287, USA; redoming@asu.edu (R.E.D.); pserhan@asu.edu (P.S.);; 4 Medical Devices and Methods Laboratory, Health Futures Center, Arizona State University, 6161 E. Mayo Blvd., Phoenix, AZ 85054, USA; 5 School of Electrical, Computer, and Energy Engineering, Ira A. Fulton School of Engineering, Tempe, AZ 85287, USA; 6 Phoenix Children’s Research Institute, Department of Child Health, College of Medicine—Phoenix, University of Arizona, 475 N. 5th St., Phoenix, AZ 85004, USA; 7 Department of Comparative Medicine, Mayo Clinic Arizona, 13400 East Shea Boulevard, Scottsdale, AZ 85259, USA

**Keywords:** urine output monitoring, urine flow meter, acute kidney injury, automated monitoring, medical device validation, point-of-care diagnostics

## Abstract

Acute kidney injury (AKI) affects thousands of hospitalized patients annually, yet early detection remains challenging as serum creatinine elevation lags behind clinical deterioration. Decreased urine output (UO) represents a key diagnostic criterion of AKI, sometimes manifesting hours before biochemical changes; however, current manual monitoring methods are labor-intensive and prone to error. Here, we developed and validated a simple, cost-effective automated urine flow meter using non-contact optical sensors, a peristaltic pump, and microcontroller-based automation for precise, real-time monitoring of urine output in clinical settings, named P-meter. Three successive prototypes (V1, V2, V3) were validated against gold-standard gravimetric measurements over 285 h of testing during animal experiments that required bladder catheterization. Iterative refinement addressed miniaturization challenges, fluid dynamics optimization, and sensor positioning to achieve progressively improved accuracy. The optimized V3 prototype demonstrated further enhanced volumetric precision, stability, and flow accuracy with near-unity linearity vs. reference method (R^2^ = 0.9889), minimal bias (mean error −0.1 mL), and 94.18% agreement within confidence limits (n = 86), outperforming the initial V1 prototype (R^2^ = 0.9971, mean error −1.69 mL, n = 207) and intermediate V2 design (R^2^ = 0.9941, mean error 3.63 mL, n = 390), primarily in terms of reduced bias and improved agreement. The P-meter offers accurate urine output monitoring at a lower cost than commercial systems, facilitating its use in early AKI detection and thereby improving patient outcomes.

## 1. Introduction

Acute kidney injury (AKI) represents a critical global health threat, affecting millions of hospitalized patients annually and contributing to approximately 2 million deaths worldwide [1,2]. Urine output (UO) and serum creatinine are the main internationally recognized diagnostic indicators of AKI [3,4,5]. However, early detection of AKI remains a major challenge because of the delayed changes in serum creatinine [6,7]. Moreover, decreased UO often manifests hours before serum creatinine and other biochemical markers change significantly, making it a particularly valuable early sign [8,9,10,11,12]. Given that even brief delays in detection can profoundly increase patient morbidity and mortality, accurate and continuous UO measurement is critical for facilitating timely clinical intervention [10,13,14,15]. Despite its clinical importance, manual UO monitoring—the current standard of care—remains inherently limited [16]. This traditional method relies on intermittent visual assessment and manual recording of urine volume from collection bags. While performed frequently (e.g., every 15 min) in critical environments like intensive care units (ICUs) and operating theaters, or less often (e.g., every 8 h) in general care settings, this manual process is labor-intensive, time-consuming, prone to error and cannot provide the continuous, high-resolution data necessary for timely detection of early oliguria (defined as a UO < 0.5 mL/kg/h for 6 h, [3,8]) and proactive clinical decision-making [17,18,19,20]. The healthcare community thus faces a critical gap between the well-established importance of precise UO monitoring for preventing adverse outcomes and the limitations of current measurement methods, specifically the lack of an affordable, accurate, and automated monitoring solution capable of continuous, real-time measurement.

Accurate automated measurement of UO presents unique technical challenges, as urine flow in healthy subjects is inherently discontinuous. This characteristic is attributed to ureteric peristalsis [21], a process in which pacemaker cells in the renal pelvis initiate waves that propagate along the ureter at a rate of approximately one to five contractions per minute [22]. Additionally, bladder catheterization creates a “dead space” within the bladder where urine must accumulate before entering the catheter drainage tube, further contributing to the discontinuous flow pattern observed in collection systems. Fluid can also become trapped within the drainage tubing by air pockets (airlocks), a frequent issue in standard catheter systems. When the tubing is manipulated, either by patient movement or clinical staff, these airlocks are disrupted, releasing the retained urine in sudden surges. This leads to transient periods of artificially low recorded urine output followed by abrupt increases once the obstruction clears. Such airlock-induced flow interruptions create measurement artifacts that reduce the accuracy and reliability of urine output monitoring. To prevent airlock formation and actively clear it when it occurs—while simultaneously preserving accurate urine output measurements and minimizing the risk of bladder suction trauma—modern systems integrate active drain-line clearance via peristaltic pumping, fail-safe catheter design incorporating multiple small-diameter lumens, and controlled venting mechanisms (at least one hydrophobic vent filter inserted along the drainage lumen) [23].

Conventional velocity-based flow meters, such as turbines or paddlewheel sensors, function effectively only under continuous or relatively uniform flow conditions and are therefore poorly suited for this application [24]. Ultrasonic flow meters, which measure fluid velocity via Doppler shift of acoustic waves, offer high accuracy but remain cost-prohibitive for widespread deployment in mass-produced medical devices [25]. Consequently, the medical device industry and academic research groups have focused on developing automated UO monitoring systems that provide continuous, high-resolution volumetric and flow-rate data, each with distinct advantages and limitations:

- Droplet-based systems: The kUO Plus (Fize Medical, formerly Nephrolog) and Urinfo 2000 (FlowSense, acquired by Baxter in 2013) employ proprietary sensing modules that detect incremental urine volume or count droplets in the drainage line. The kUO Plus provides microliter-resolution measurements with continuous flow-rate calculation and alarm functionality [26], whereas the Urinfo 2000 utilizes a dual-chamber system with infrared drop detection to convert drop frequency into flow rates [27]. Both systems have undergone clinical validation and interface with ICU monitoring systems [19,28,29].
- Active drainage systems: The FDA-approved Accuryn Monitoring System (Potrero Medical) represents a sophisticated, multi-parameter approach that uses an actively drained, dual-lumen catheter with controlled, low-vacuum pumps to maintain continuous drainage and eliminate trapped air through automated drain line clearance every 5 min. The system integrates pressure transducers and acoustic/ultrasound sensors to measure not only urine flow rate and cumulative volume but also intra-abdominal pressure and core body temperature in real-time. A recent multicenter study of cardiac surgery patients demonstrated that the Accuryn AKI Alert system diagnosed AKI an average of 33 h earlier than standard serum creatinine measurements [30]. The Accuryn Registry is an ongoing multicenter study enrolling cardiovascular surgery patients to analyze physiological data and its relationship with AKI [31].
- Thermal and capacitive methods: ClarityRMS (RenalSense) employs a cost-effective thermal approach, applying thermal energy pulses to flowing urine and calculating flow rate based on temperature differentials. Clinical trials demonstrated significant accuracy with a mean volume difference of only 2.29 mL (*p* = 0.61) compared to gravimetric measurements [18,32]. Research prototypes by Otero utilize capacitive sensing with magnetic drainage mechanisms [17], while systems by Padilla employ infrared sensors along drain tubes [33], and Piyapema’s design uses thermistor-based droplet detection with IoT connectivity [34].
- Developmental systems: Serenno Medical’s Sentinel system, currently under development, has completed initial trials demonstrating 96% accuracy over 1300 h of monitoring with more than 40 patients [20]. Art Medical has patented a droplet-counting system that uses fast-gated cameras to estimate the volumes of individual drops [35].

A summary of representative commercial, developmental, and research-stage automated urine output monitoring systems and their validation status is provided in Supplementary Table S1.

The development of automated UO monitoring aims to address the limitations of error-prone manual measurement, particularly in critical care settings [30]. Current commercial and research devices leverage several distinct technologies, ranging from high-resolution droplet detection to multi-parameter physiological sensing systems. While these innovations represent significant technological advances, substantial barriers persist between clinical needs and the widespread adoption of clinical practices. Many promising automated urine measurement systems validated in research settings have failed to achieve commercialization due to regulatory complexity, manufacturing challenges, or the high costs associated with regulatory approval pathways [36]. Commercial systems that have achieved regulatory clearance often require sophisticated, multi-component infrastructure, which increases operational complexity, error probability, and the need for extensive staff training [17,37]. High-reliability systems usually incur substantial per-unit costs, which limit their widespread adoption, particularly in resource-constrained healthcare settings where cost-effectiveness is a critical determinant of technology adoption [38]. Furthermore, several promising technologies remain in development or in the clinical testing phase without clear pathways to market deployment. The healthcare community requires an improved solution that delivers reliable, real-time UO measurements while maintaining operational simplicity, cost-effectiveness, and seamless integration with existing hospital infrastructure. Such a device must require minimal staff training, provide measurement accuracy comparable to existing gold standards, and ultimately contribute to improved patient monitoring and clinical outcomes.

This paper presents the development and validation of the P-meter, an accurate, simple, and cost-effective automated urine flow meter designed to address these unmet clinical needs through a novel approach that combines non-contact optical sensing, peristaltic pumping, and microcontroller-based automation. We demonstrated that a simplified design, prioritizing ease of use, manufacturing scalability, and affordability, could achieve measurement accuracy comparable to that of existing commercial systems while enabling broader clinical deployment. Three iterative prototypes (V1, V2, and V3) underwent refinement and systematic validation against gold-standard gravimetric methods. The specific objectives of this study were to (1) design and refine an automated urine monitoring system addressing limitations of existing technologies; (2) validate measurement accuracy, precision, and reliability against gold-standard gravimetric methods with urine specific gravity corrections; and (3) evaluate the device’s potential for clinical deployment through systematic assessment of operational performance across multiple prototype iterations.

## 2. Materials and Methods

### 2.1. Principle of Operation

**Figure 1:**
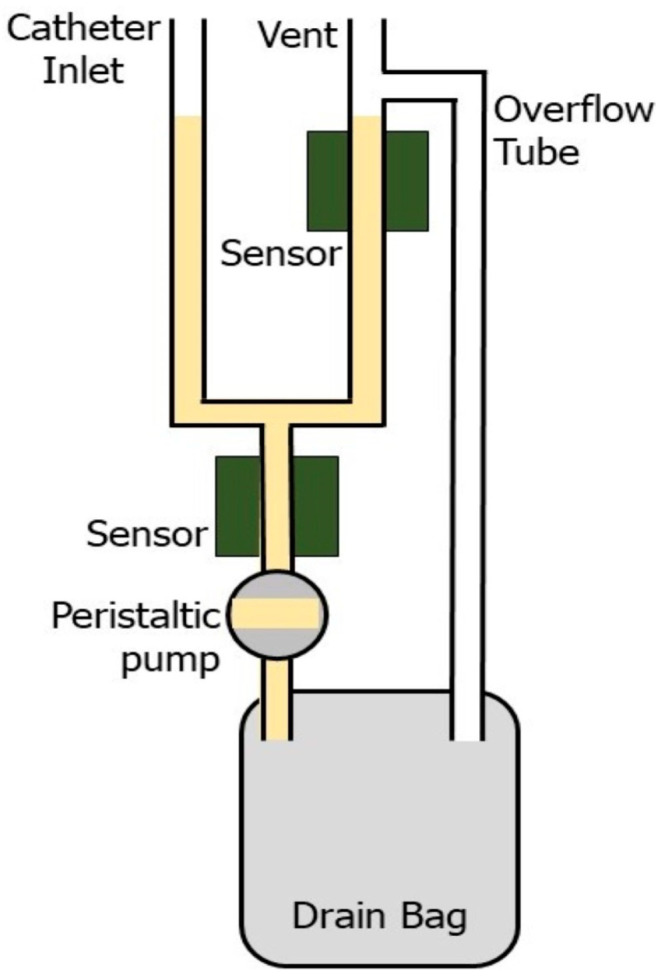
Schematic of the P-meter.

Figure 1 presents the schematic design of the “P-meter”, an automated urine flow measurement system developed for continuous, real-time volumetric monitoring. The device integrates seamlessly with standard urinary catheter drainage systems through a dedicated catheter inlet port positioned at the device’s proximal end. The P-meter comprises the following primary functional components: a graduated measurement chamber with a catheter inlet and an atmospheric vent, dual optical sensors positioned at predetermined volumetric thresholds, an atmospheric vent, a calibrated peristaltic pump, a standard urine drain bag, an overflow tube, and a microcontroller-based control system (Figure S4).

The catheter inlet provides a secure, fixed connection to the distal end of a standard catheter drain tube. Urine flows naturally by gravity from the bladder through the catheter system into the P-meter’s measurement chamber via rigid fluid lines. The measurement chamber incorporates two non-contact optical sensors positioned at predetermined vertical intervals to define a fixed measurement volume. As urine accumulates within the collection chamber/tubes, the liquid level rises until it reaches the upper optical sensor, which generates a signal change that triggers the microcontroller to activate the peristaltic pump. The pump operates at a pre-calibrated, constant volumetric flow rate, vacating the contents of the collection chamber and transferring the measured volume to the drain bag. When the urine sample drops below the lower sensor threshold, the microcontroller receives a second signal that terminates pump operation, allowing the measurement cycle to repeat.

The volumetric thresholds were selected based on physiologically relevant urine excretion rates to balance measurement resolution and system stability; volumes that were too small resulted in continuous pump activation, whereas larger volumes increased chamber size and reduced sensitivity to low urine output. Accordingly, threshold volumes in the range of approximately 7.5–25 mL were implemented across prototype versions.

Volumetric calculation is performed using the time-flow integration method, in which the microcontroller records the precise pump activation duration (*t*) and calculates the dispensed volume (*V*) as *V* = *Q* × *t*, where *Q* denotes the known, calibrated pump flow rate. This approach eliminates the need for direct volume measurement, providing continuous and automated quantification of urine output. Urine flow rate is calculated as a derived parameter by dividing the measured transferred volume by the corresponding time interval between successive pump activation events. No additional smoothing or averaging is applied beyond the intrinsic integration imposed by the volumetric measurement cycle.

The measurement chamber incorporates several critical safety features. To address regulatory requirements for processing medical devices that handle bodily fluids, the design utilizes non-contact optical sensors rather than direct fluid-contact measurement. This approach eliminates the need for sterilizing electronic components and simplifies the fluid path for cleaning validation. Components in direct liquid contact (measurement chamber, tubing, and drainage bag) are designed as potentially disposable, single-use assemblies. The entire plumbing system maintains continuous atmospheric venting to prevent pressure accumulation within the drainage pathway, thereby eliminating potential suction traumas to bladder walls. Finally, an overflow bypass tube provides a fail-safe drainage mechanism; if sensors or the pump malfunction, the overflow tube drains the collected liquid by gravity, preventing retrograde flow or system overfilling. The fluid accumulation chamber includes a small, ported connection for future integration with a chemical biomarker testing subsystem.

The subsequent subsections provide detailed specifications for the principal functional subsystems: optical sensors, peristaltic pump system, and microcontroller hardware/software architecture.

### 2.2. Flow Rate and Liquid Detection Sensor

Various transducers can detect flow rates or the presence of liquids. These sensors generally fall into two categories: contact-based (e.g., resistive, tuning fork, float switches, electromagnetic) and non-contact (e.g., capacitive level detection, ultrasonic transducers, photoelectric/optical). Contact-based sensors for bodily fluids require either single-use sensors or sterilization between patients, making them impractical due to cost considerations.

Ultrasonic systems offer high versatility and precise, non-contact measurements for detecting fluid levels or volumetric flow rates. An ultrasonic transmitter emits waves that reflect off the fluid, and the receiver processes these signals to calculate the flow. While highly accurate, this technology was excluded because the measurement architecture and associated electronics require substantial development, thereby increasing both the cost and the time. The high cost of commercially available ultrasonic products made them impractical for the P-meter’s final design.

Optical level-detecting sensors operate by emitting infrared light from LEDs or laser beams on one side of the measurement medium and detecting photons on the opposite side with high-energy photodiodes. These sensors offer advantages in high-pressure and high-temperature applications. They are compact, contain no moving parts, and are resistant to electromagnetic interference. Many traditional optical sensors require direct contact with the process liquid. To avoid the high cost and impracticality of single-use sensors or the need for sterilization between patients, a critical consideration for bodily fluids, a liquid tube sensor configuration was necessary. This configuration, based on similar principles, contains the fluid within a transparent tube, with the emitter-detector positioned externally.

Based on the requirements for non-contact measurement, simplicity, and low cost, a liquid tube sensor was selected. Among commercially available products from manufacturers such as TT Electronics and Panasonic, the TT Electronics OPB350 series (Surrey, UK) was chosen for its compatibility with larger tube diameters, which are suitable for urine drainage lines. The OCB350 variant includes reference circuitry, providing logic output with automatic estimation capability and preset trip positions. Figure S1 displays the OCB350L250Z sensor module, which accommodates tubes with a 1/4-inch outside diameter. This ready-to-use module features six lead wires: two for power connection, one for providing analog photodiode output, two for logic outputs to detect tube and liquid, and one for calibration. Four onboard indicator LEDs signal device status: green for successful calibration, red for calibration failure, blue when the analog gain exceeds the calibration point, and green when it is below the calibration point. Response sensitivity can be adjusted by repositioning the shorting bar to change the internal phototransistor load resistance [39].

Test data was acquired using an Arduino Mega 2560 (Arduino LLC, Monza, Italy) from three output pins: analog output voltage, Logic Out A, and Logic Out B. The testing utilized a standard 1/4-inch OD tube inserted into the OCB350 sensing module. Calibration is performed by grounding the sensor calibration pin to the microcontroller. Following calibration, the analog voltage resets to a new baseline serving as a reference for logic outputs. Logic Out A detects tube presence and maintains a consistent high state (≈approximately 1 V) after calibration. Logic Out B serves as the primary liquid sensing output in the P-meter setup. When liquid passes through the tube, Logic Out B transitions from high (≈1 V) to low (≈0 V). The analog voltage correspondingly changes from ≈2.49 V without liquid to ≈4.73 V with liquid present. Sensor response occurs within milliseconds. This module was selected for the P-meter due to its simple and cost-effective implementation, with rapid setup capability. Figure S2 displays the acquired test data.

### 2.3. Fluid Transfer and Volumetric Measurement: Peristaltic Pump

The P-meter requires a mechanism to safely and precisely transfer urine from the collection chamber to the waste bag while simultaneously calculating the discharged volume. Peristaltic pumps were selected for this function because they transport fluids by compressing flexible tubing with rollers rotating on a drum. This design is critical in a biomedical context, as it contains the fluid entirely within the tubing, eliminating the risk of exposure to contamination and the need for pump sterilization. This makes them ideal for clean, sterile, or highly reactive fluids.

After evaluating various laboratory-scale options, the Atlas Scientific EZO-PMP dosing pump (Figure S3) was selected for its compact size, cost-effectiveness, and straightforward integration with a microcontroller via its Universal Asynchronous Receiver/Transmitter (UART) protocol. The EZO-PMP is a compact peristaltic dosing pump measuring approximately 38 mm in diameter and 100 mm in length, featuring an integrated DC motor driver and UART serial controller. Designed for 5 mm outer diameter tubing, it dispenses fluids at flow rates from 0.5 to 100 mL/min with ±1% accuracy across this range. The pump supports four operational modes: (i) continuous dispensing, (ii) volumetric dispensing, (iii) constant flow rate, and (iv) dose-over-time delivery. These modes enable fluid handling across a range of experimental conditions while maintaining repeatable operation. The device interfaces with any TTL UART connection, including FTDI USB bridges or Arduino RX/TX lines, and supports I^2^C communication. An onboard RGB LED provides color-coded operational status indicators. Each unit operates with 3.3–5 V logic and requires 12–24 V for motor operation [40].

### 2.4. System Control: Microcontroller

An embedded microcontroller is essential for managing sensors and actuator functions within the P-meter system. Microcontrollers are compact integrated circuits comprising a Central Processing Unit (CPU), input-output (I/O) ports, Random-Access Memory (RAM), Read-Only Memory (ROM), and an oscillator, explicitly designed for application-specific tasks. Among commercially available microcontrollers, the Arduino Mega 2560 (Figure S4) was selected for P-meter development due to its favorable learning curve, ease of programming, and cost-effectiveness.

The Arduino Mega 2560 is an open-source microcontroller board based on the ATmega2560. The Mega provides many digital and analog input/output pins. Crucially, it offers multiple hardware serial ports for connecting sensors, actuators, and other devices, which are necessary for simultaneous, reliable communication with various components, specifically the EZO-PMP peristaltic pump (UART) and other potential expansion modules. The board can be powered via USB or an external source (7–12 V recommended). Programming utilizes the Arduino Integrated Development Environment (IDE) with the Wiring language, a simplified C/C++ variant designed for physical computing applications [41]. Its capacity to handle complex I/O and communication protocols ensures it can effectively manage continuous data acquisition from the optical sensor and control commands for the peristaltic pump, thereby meeting the core requirements of the P-meter system.

### 2.5. Calibration and Volumetric Correction

The liquid level sensor utilizes manufacturer-provided calibration with digital control, eliminating the need for additional adjustment. However, obtaining accurate volumetric readings and derived flow rates required linear correction of the collective system. Preliminary testing revealed that the device consistently reported volumes that were slightly higher than the actual values, with the magnitude of this error varying across the measurement range. To precisely characterize the error pattern and establish the necessary linear correction, test volumes ranging from 1 to 100 mL were passed through the P-meter device. The actual reference volumes were determined using two methods for verification: (a) volumetric measurement with graduated measuring cylinders and (b) gravimetric measurement using liquid weight.

#### Addressing Low-Volume Pumping Threshold

A challenge arose from the system’s operational design: the peristaltic pump discharges only after the fluid reaches the upper-level sensor. This threshold volume was approximately 25 mL in Prototype Versions 1 and 3, and 7 mL in Prototype Version 2. To accurately characterize error patterns for sub-threshold volumes, a manual triggering approach was employed. In this method, fixed, known liquid volumes were carefully introduced near the upper optical sensor through the venting port, causing the sensor to detect a full condition and initiate the pumping cycle, thereby allowing accurate measurement of volumes below the normal activation threshold. Using this approach, two linear calibration relationships (*y* = *mx* + *c*) were established: one derived from standard measurements spanning the full operational volume range and a second derived specifically from the low-volume, manually triggered measurements. Application of these calibration curves ensured continuity across the full measurement range and enabled the final reported output to accurately reflect the true transferred volume.

### 2.6. Experimental Methodology

The P-meter prototypes underwent evaluation during renal physiology experiments that required the use of a bladder catheter. Experiments were designed with 20-min collection intervals, although actual sampling times occasionally varied due to the experimental workflow. Urine samples were analyzed at room temperature, in the device next to the animal. Most validation sessions lasted 6 h to ensure the evaluation of the P-meter’s stability and accuracy over prolonged clinical simulation periods. The device was integrated directly into the catheter drainage line, replacing the standard urine collection bag. The protocol focused on validating the P-meter’s calculated metrics against precise reference measurements over extended periods.

The P-meter transmitted time-stamped serial text streams containing device-calculated UO metrics (cumulative volume and flow rate) via USB. Data capture utilized the PuTTY terminal emulator, with output saved as plain-text files for offline analysis and review. To establish a highly accurate reference standard, all urine exiting the P-meter was collected in pre-weighed containers. The net urine mass was calculated by subtracting the weight of the empty container from the weight of the filled container. To avoid assuming a density of 1 g/mL, the urine specific gravity (USG) of each 20-min collected aliquot was measured using a refractometer. USG represents the ratio of sample density to water density at the measurement temperature. Sample density was calculated as: urine density = USG × water density, and reference volume was computed as: volume = mass ÷ urine density. Both mass and USG values were recorded for each aliquot.

### 2.7. Validation Testing Conditions

Validation experiments were conducted during renal physiology studies in anesthetized animal subjects. The Mayo Clinic approved an IACUC (A00006128) for this project to conduct animal studies. The V1 prototype was evaluated across 13 experiments totaling 79 h of operation, the V2 prototype underwent testing in 30 experiments spanning 176 h, and the V3 prototype was evaluated in 5 experiments totaling 30 h of operation. Experiments with incomplete measurements due to sensor malfunctions or physiological complications were excluded from analysis.

## 3. Results

**Figure 2:**
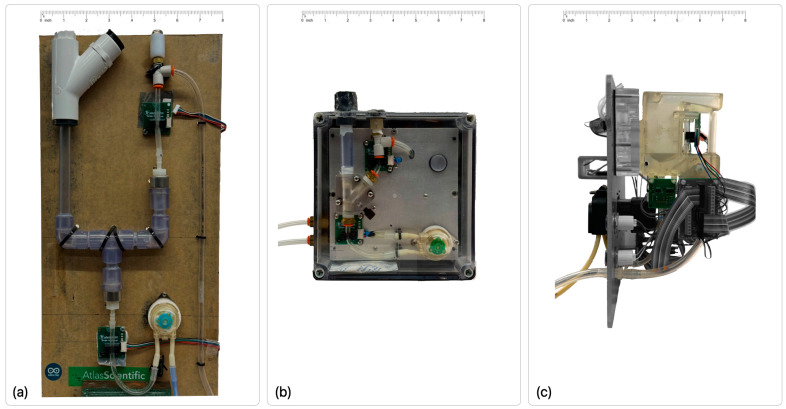
Evolutionary development of the P-meter automated urine monitoring system. Each version features two OCB350 tube liquid sensors, a collection chamber, and a peristaltic pump. (**a**) P-meter Version 1 (V1) proof-of-concept prototype demonstrating integration with a standard urinary catheter drainage system; (**b**) P-meter Version 2 (V2) plug-and-play prototype with a significantly smaller footprint, enclosed within an IP52-rated housing; (**c**) P-meter Version 3 (V3) prototype illustrating an optimized collection chamber geometry and a modular interface for integration with a chemical analyzer (under development, shown in contrasting color). V3 also incorporates an upgraded peristaltic pump (Boxer 9QQ, **Boxer GmbH, Ottobeuren, Germany**) and a custom-integrated hydrophobic membrane venting system.

P-meter Prototype Development. Three successive prototype generations (P-meter V1, V2, and V3) were developed through an iterative design process that focused on addressing performance limitations identified during validation (animal experiments/physiological) testing. The evolution from the initial proof-of-concept (V1) through the intermediate prototype (V2) to the final optimized system (V3) is illustrated in Figure 2, with the key hardware differences across versions summarized in Supplementary Table S2.

### 3.1. P-Meter Version 1 (V1)

#### 3.1.1. Design and Characteristics

The P-meter V1 prototype served as the initial proof-of-concept device, constructed using off-the-shelf components mounted on a simple mechanical platform for its first deployment in renal physiology experiments (Figure 2a). The V1 system featured a 25 mL measurement chamber capacity and operated with a measurement threshold of approximately 25 mL before pump activation.

#### 3.1.2. Calibration and Linear Correction

The initial V1 prototype exhibited a systematic volumetric overestimation, necessitating linear calibration correction. To characterize this error, reference volumes ranging from 1 to 100 mL were established using gravimetric measurements (analytical scale, ±0.01 g precision) combined with urine specific gravity determinations. A notable challenge was the sensor activation threshold of 25 mL. To analyze sub-threshold errors, a manual triggering method was employed by introducing fluid through the atmospheric vent rather than the standard catheter inlet. Linear regression analysis identified a consistent proportional error relationship, and the resulting correction equation was applied to all subsequent data. Post-calibration ex situ validation testing (n = 3 replicate experiments) confirmed the accuracy of this correction, yielding an exceptionally high correlation coefficient.

#### 3.1.3. Validation and Performance Analysis

**Figure 3:**
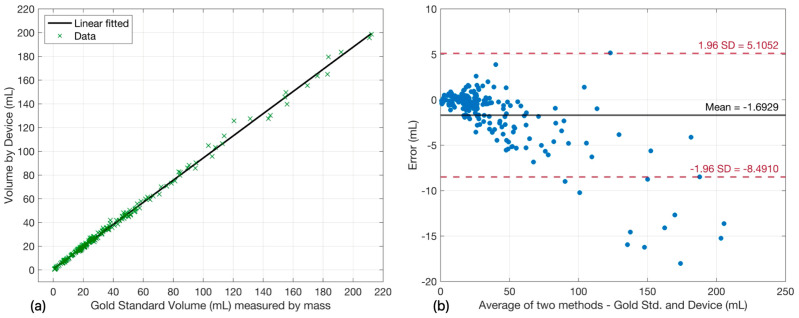
Performance validation of P-meter V1 prototype. (**a**) Linear correlation analysis: V1-measured volume versus gravimetric reference volume (n = 207 measurements from 13 experiments; 79 h total operation). Linear regression: y = 0.9346x + 0.9185, R^2^ = 0.9971. The solid line represents a linear fit. (**b**) Bland–Altman analysis: Difference between measured and reference volumes (*y*-axis) plotted against mean volume (*x*-axis). Solid horizontal line indicates mean bias (−1.69 mL); dashed lines represent 95% limits of agreement (mean ± 1.96 SD). Twelve outliers (5.80%) exceeded the 95% confidence interval.

Figure 3a presents the correlation between V1-measured volumes and gravimetric reference values (n = 207 measurements). Linear regression analysis yielded: y = 0.9346x + 0.9185 with R^2^ = 0.9971, indicating strong linearity. The regression slope of 0.9346 represents a 6.54% proportional underestimation, while the interception of ~0.92 mL indicates minimal systematic offset.

Bland–Altman analysis (measurement error versus 2-methods average volumes, Figure 3b) revealed a mean bias of −1.69 mL. The 95% confidence bounds, calculated as mean ± 1.96 × SD, encompassed 195 of 207 measurements (94.20%). Twelve measurements (5.80%) fell outside the 95% confidence interval.

### 3.2. P-Meter Version 2 (V2)

#### 3.2.1. Design Refinements

The V2 prototype incorporated significant miniaturization and functional enhancements (Figure 2b). The physical dimensions were reduced to 175 mm × 175 mm × 100 mm, with a total system weight of 1.1 kg. The device achieved an IP52 Ingress Protection rating [42], providing protection against dust infiltration and dripping liquid. The key design modifications in V2 included: (1) reduction of measurement chamber capacity from 25 mL to 7.5 mL to increase measurement frequency; (2) implementation of plug-and-play functionality with automatic sensor calibration upon power-up; (3) integration of serial port communication for real-time data transmission; and (4) addition of a downstream urine chemical analysis sampling port within the collection chamber for future biochemical analysis capabilities to enhanced functionality.

#### 3.2.2. Calibration and Linear Correction

Similar to its predecessor, the V2 prototype also required a linear calibration to account for systematic volumetric overestimation. The same gravimetric and specific gravity methods were used to establish reference volumes ranging from 1 to 100 mL. However, the reduced liquid-holding capacity of V2 lowered the sensor activation threshold to 7 mL. This change required the manual triggering method (fluid introduced via the atmospheric vent) to be applied only for volumes below the significantly lower than the 7 mL threshold. After deriving and using the linear correction equation, post-calibration ex situ validation testing (n = 3 replicate experiments) confirmed the successful adjustment, yielding a very high correlation coefficient.

#### 3.2.3. Validation and Performance Analysis

**Figure 4:**
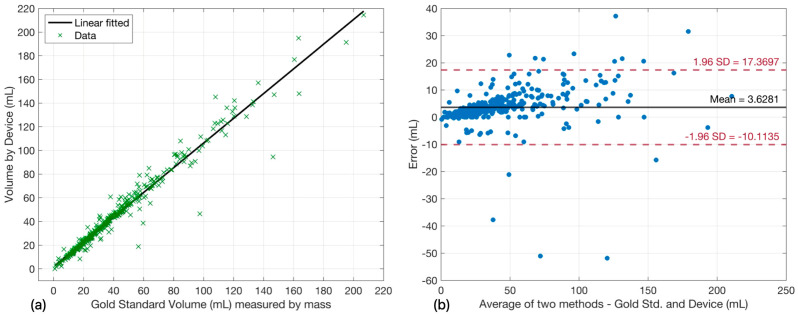
Performance validation of P-meter V2 prototype. (**a**) Linear correlation analysis: V2-measured volume versus gravimetric reference volume (n = 390 measurements from 22 experiments; 130 h total operation). Linear regression: y = 1.0446x + 1.7856, R^2^ = 0.9613. The solid line represents a linear fit. (**b**) Bland–Altman analysis: Difference between measured and reference volumes plotted against mean volume. Solid horizontal line indicates mean bias (+3.63 mL); dashed lines represent 95% limits of agreement. Fourteen outliers (3.59%) exceeded the 95% confidence interval.

Despite design improvements, the V2 prototype demonstrated reduced accuracy compared to the V1 prototype. Figure 4a shows the correlation between V2-measured volumes and reference values across 22 experiments (n = 390 measurements; 130 total operating hours). Linear regression yielded: y = 1.0446x + 1.7856 with R^2^ = 0.9613. The regression slope of 1.0446 indicates 4.46% proportional overestimation, while the intercept of 1.79 mL represents a greater systematic offset than V1.

The corresponding Bland–Altman analysis (Figure 4b) revealed a mean bias of +3.63 mL with 95% confidence bounds encompassing 376 of 390 measurements (96.41%). Fourteen outliers (3.59%) exceeded the 95% confidence bounds.

### 3.3. P-Meter Version 3 (V3)

#### 3.3.1. Design Modifications and Optimization

Analysis of V1 and V2 performance limitations identified several critical deficiencies: air bubble entrapment, residual fluid accumulation in flow paths, particulate debris obstruction, inadequate atmospheric venting, and pump flow rate variability. The V3 prototype incorporated targeted engineering solutions to address these issues (Figure 2c).

Primary design modifications included: (1) expansion of measurement chamber volume to 25 mL; (2) stereolithography (SLA) 3D-printed chamber with optimized internal geometry and surface smoothness to minimize fluid retention; (3) threaded catheter inlet connector to ensure hermetic sealing; (4) custom high-flow PTFE hydrophobic membrane vent (mounted in 3D-printed bracket) to enhance atmospheric equilibration while maintaining sterility; (5) replacement of peristaltic pump with Boxer 9QQ model [43] featuring integrated Pico driver and four-roller mechanism for improved dispensing accuracy and flow stability; (6) an upgraded Microchip PIC32 microcontroller substituting the Arduino Mega to improve timing control, reliability, and system integration; and (7) addition of manifold port for potential integration with biochemical analysis systems. The overall concept and operating principle remain unchanged, with improvements in the hardware.

#### 3.3.2. Validation and Performance Analysis

**Figure 5:**
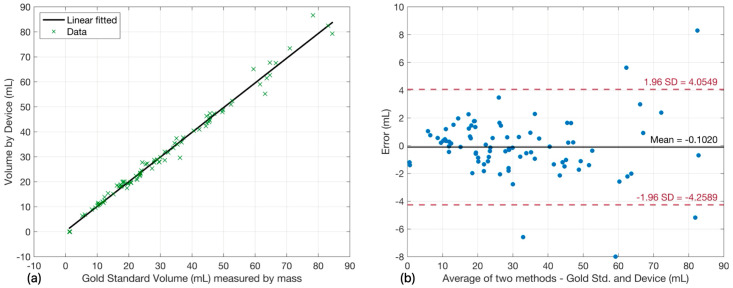
Performance validation of the final P-meter V3 prototype demonstrates superior accuracy. (**a**) Linear correlation analysis: V3-measured volume versus gravimetric reference volume (n = 86 measurements from 5 experiments; 30 h total operation). Linear regression: y = 0.9889x + 0.2487, R^2^ = 0.9881. The solid line represents a linear fit showing near-perfect agreement (slope = 0.9881, approaching unity). (**b**) Bland–Altman analysis: Difference between measured and reference volumes plotted against mean volume. Solid horizontal line indicates mean bias (−0.10 mL); dashed lines represent 95% limits of agreement. Five outliers (5.81%) exceeded the 95% confidence interval.

Experimental validation of the V3 prototype was conducted across five renal physiology experiments totaling 30 h of operation (n = 86 measurements. V3 represents a recent development, where this initial validation was necessarily limited in scope due to the availability of surgical resources; however, expanded studies are planned). Figure 5a presents the correlation between V3-measured and reference volumes. Linear regression analysis yielded: y = 0.9889x + 0.2487 with R^2^ = 0.9881, demonstrating excellent linearity approaching ideal unity slope (slope deviation: −1.11%). The intercept of 0.25 mL represents a substantially reduced systematic offset compared to previous versions.

Bland–Altman analysis (Figure 5b) revealed a mean bias of −0.10 mL, representing a 17-fold improvement compared to V1 (mean bias: −1.69 mL) and 37-fold improvement compared to V2 (mean bias: +3.63 mL). The 95% limits of agreement encompassed 81 of 86 measurements (94.19%), with five outliers (5.81%) exceeding confidence bounds. The V3 mean absolute bias of 0.10 mL is clinically negligible for UO monitoring applications, where hourly output typically ranges from 30 to 100 mL in euvolemic (normal fluid balance) patients.

## 4. Discussion

### 4.1. Comparative Performance and Design Advantage of V3

This study presents the development and rigorous validation of the P-meter, a novel automated UO monitoring system designed to address critical gaps in the detection of AKI and other related diseases. Through three iterative prototype generations, we achieved progressive improvements in measurement accuracy, culminating in the V3 device, which demonstrated exceptional performance with a slight mean bias (−0.10 mL) and an excellent correlation (R^2^ = 0.9881) with gravimetric reference standards, the gold standard methodology. While R^2^ was used to assess linearity, comparative prototype performance was primarily evaluated using systematic bias, regression slope, and limits of agreement, which more directly reflect volumetric accuracy for urine output monitoring.

The demonstrated volumetric accuracy of V3 compares favorably with that of clinically validated automated urine monitoring devices reported in the literature, positioning it as a competitive and cost-effective solution. The final prototype achieved a mean bias of −0.10 mL across 86 measurements, showing exceptional agreement with gravimetrically corrected reference volumes. This level of precision exceeds that of several established benchmark systems. For instance, the ClarityRMS system (RenalSense), which employs thermal flow measurement technology, demonstrated a mean volume difference of 2.29 mL (*p* = 0.61) in clinical trials [12]. While this thermal approach is economic, the P-meter V3 shows a 23-fold lower mean bias (0.10 mL vs. 2.29 mL), suggesting superior volumetric precision for applications requiring high-resolution AKI detection. Similarly, when compared to the Urinfo 2000 system (FlowSense/Baxter), which utilizes infrared droplet detection, it exhibited an 8% mean relative error (approximately ±25 mL calculated error) relative to cylinder measurements [19]. The bias of V3 exhibits tighter agreement across the measurement range, particularly for low-volume measurements, which are essential for early detection of oliguria [12]. Gravimetric scale-based systems have demonstrated mean differences of −6.31 ± 15.03 mL/h compared to reference cylinders [44]. Even when compared with gravimetric scale-based systems, the P-meter’s volumetric integration method provides equivalent accuracy, with the added advantage of continuous flow-rate calculation without dependence on external scales.

This excellent precision of V3 is due to its architectural features. The system employs a volumetric integration methodology that directly measures accumulated volume rather than inferring it from flow rates or droplet counts, thereby avoiding the compounding errors inherent in derivative approaches. Furthermore, the use of a calibrated, stepper-motor-driven peristaltic pump ensures precise volumetric delivery characteristics with minimal pulsation effects, a common source of artifact in other pumping technologies. Finally, the closed-system architecture is critical for maintaining measurement integrity over extended monitoring periods, eliminating the evaporative losses that compromise the accuracy of open-measurement systems in clinical environments. Consequently, V3 is positioned as a robust, accurate, and stable device for continuous UO monitoring.

### 4.2. Design Evolution

V1 Prototype: Proof-of-Concept Validation. The V1 prototype successfully demonstrated the fundamental feasibility of the dual-chamber, photo-interrupter-based measurement approach, achieving a high correlation coefficient of R^2^ = 0.9971 against the gravimetric gold-standard method. However, the relatively large mean bias (−1.72 mL) and moderate percentage of measurements falling outside 95% confidence limits (6.28%) identified opportunities for refinement. Its 25 mL measurement threshold, while suitable for proof-of-concept validation, showed low resolution for detecting AKI.

V2 Prototype: Miniaturization Challenges. The V2 prototype successfully incorporated substantial miniaturization and improved component integration, reducing the physical footprint for better bedside suitability. However, these changes resulted in degraded performance (mean bias: −2.29 mL; R^2^ = 0.9941; only 89.40% within 95% CI) compared to V1.

Post-validation analysis identified four primary technical factors that contributed significantly to the outcome. First, the reduced chamber volume amplified the relative impact of peristaltic pump pulsations, creating transient meniscus oscillations that intermittently disrupted photo-interrupter signal stability during fluid transfer. Second, the effects of meniscus geometry became more pronounced; the narrower chamber increased the surface tension-to-volume ratio, leading to greater curvature variations and systematic errors near volume thresholds. Third, the compact V2 architecture required tighter sensor positioning tolerances, as minor positional variations compromised the optical path geometry, thereby reducing measurement repeatability. Finally, the miniaturized chambers retained proportionally larger residual fluid volumes after pump evacuation, introducing cumulative accounting errors over multiple measurement cycles. These findings highlight the intricate interplay between component miniaturization, fluid dynamics, and measurement accuracy—a challenge often underestimated in the development of discontinuous-flow volumetric systems.

Lessons from V1 and V2 Development. Beyond the performance-specific issues of V1 and V2, testing revealed several key design challenges that necessitated V3 for clinical viability:

- Fluid Dynamics and Contamination: Air bubble entrapment within fluid lines generated false sensor signals. This was related to surface-induced bubbles from the internal thread surface and required designing flow paths to allow smooth trickling rather than turbulent entry. Debris obstruction and fluid accumulation at the flat bottom of the wye fitting required installing an inlet filter and tapering the outlet for complete drainage.
- System Integrity and Reliability: The catheter connection lacked a secure seal, causing overflow leakage, necessitating a clamping lock mechanism. Inadequate venting created internal vacuum conditions, exacerbating inlet leakage. Blood cells in urine were also observed to impair sensor function (interference), indicating the need for further investigation into optical compatibility, specifically the sensor’s behavior in the presence of blood, proteins, and sediments.
- Component and Manufacturing Constraints: The current design does not facilitate efficient production-scale manufacturing and assembly, and the necessary component replaceability (disposable plumbing: tubes and fittings) is essential for hospital applications. Furthermore, the existing peristaltic pump exhibited variable accuracy and reliability, suggesting that a stepper motor-driven peristaltic pump merits investigation for enhanced measurement precision.

The development of V3 systematically addressed the performance limitations identified in V2 through targeted engineering interventions. These refinements focused on stabilizing the fluid dynamics and tightening manufacturing precision:

1. Optimized Chamber Geometry: The chamber diameter was increased, while the overall volume threshold was maintained through height adjustments. This change achieved an optimal balance between miniaturization and meniscus stability, countering the surface tension effects observed in V2.
2. Enhanced Pump Control: New algorithms were implemented to control the stepper motor, including ramped acceleration/deceleration profiles. This modification significantly reduced fluid pulsation amplitude, minimizing meniscus disturbances during transfer operations.
3. Improved Sensor Mounting: The integration of precision-machined sensor brackets with defined alignment features reduced photointerrupter positional variability. This ensures consistent optical path geometry across manufacturing batches, improving measurement repeatability.
4. Residual Volume Compensation: Calibration protocols were updated to include empirical characterization of residual volumes under various viscosity conditions, enabling software-based correction during regular operation.

The V3 device’s final performance highlights its superior precision and robustness, as evidenced by the regression slope and a near-zero intercept, indicating near-ideal agreement between the measured and reference volumes. The mean error confirms a substantially reduced measurement bias across the measurement range. This strong performance confirms the V3 as a novel device capable of measuring real-time UO with high reliability and accuracy, positioning it as a promising candidate for clinical deployment. Table 1 presents a comparison of validation and performance parameters for the V1, V2, and V3 prototypes.

### 4.3. Clinical Significance

#### 4.3.1. AKI Detection Sensitivity

The KDIGO criteria for AKI diagnosis require measurement of UO with sufficient sensitivity to detect oliguria (<0.5 mL/kg/h over 6-h windows) [3]. For a 70 kg patient, this threshold corresponds to approximately 35 mL/h or 210 mL over 6 h. Traditional hourly manual measurements introduce ±10–15 mL measurement uncertainty due to timing imprecision, visual estimation errors, and incomplete drainage. The P-meter V3’s −0.10 mL mean bias represents < 0.3% relative error for typical hourly volumes (30–100 mL range), substantially improving detection sensitivity for borderline oliguria. The 94.19% of measurements falling within 95% confidence limits demonstrates reliability comparable to laboratory-grade volumetric standards, enabling confident differentiation between true oliguria and measurement artifact.

#### 4.3.2. Fluid Management Precision

Critically ill patients frequently require hourly fluid balance calculations to guide resuscitation, diuretic therapy, and renal replacement therapy initiation decisions [15]. Current practice tolerates cumulative errors of ±50–100 mL/h from combined input/output measurement inaccuracies. The P-meter’s sub-milliliter precision could enable tighter fluid management protocols, potentially reducing complications associated with both fluid overload (pulmonary edema, prolonged mechanical ventilation) and inadequate resuscitation (persistent AKI, organ hypoperfusion).

#### 4.3.3. Research Applications

Beyond direct clinical care, the P-meter’s measurement precision enables research applications that were previously limited by the accuracy of output monitoring. For instance, pharmacokinetic studies benefit from precise urine collection intervals, which improve the accuracy of drug clearance calculations and renal elimination modeling. Another example is for biomarker validation, where accurate volume documentation reduces confounding in urine biomarker concentration studies, particularly for low-abundance analytes. A final example is from physiological research: High-resolution UO patterns may reveal subtle physiological responses to interventions (such as fluid challenges and vasoactive medications) that are currently obscured by measurement noise.

### 4.4. Limitations and Future Directions

Despite the superior performance of V3, several limitations remain. The study involved a smaller sample size for the V3 prototype than V1 and V2, reflecting its role as an initial validation stage constrained by available animal surgical resources, with no human testing or direct comparison to commercial urine monitoring systems. Nevertheless, all V3 measurements were obtained under identical protocols and validated against gold-standard gravimetric measurements using urine-specific gravity correction, supporting the internal consistency of the results. Because flow rate is derived from gravimetrically validated volumetric measurements and time stamps, flow accuracy is inherently constrained by volumetric accuracy and timing precision rather than by a separate flow sensor. Long-term stability and potential sensor drift over extended operation were not assessed, and performance with pathological urine samples (hematuria, pyuria, crystalluria) remains unverified. Regulatory considerations and clinical implementation pathways also require further evaluation.

Future work will focus on large-scale validation involving thousands of hours (greater than 3000 measurements) of real-time operation in clinical settings to assess repeatability, stability, and performance under variable flow conditions. Manufacturing will be optimized through design-for-manufacturing principles, employing modular, single-patient disposable components (collection chamber and peristaltic tubing) paired with a reusable optical sensing unit for cost efficiency and sterility. Integration with hospital monitoring networks via digital and IoT interfaces will enable automated data logging, trend analysis, and early alerts for AKI. Subsequent versions will explore multi-analyte sensing via in-line biochemical analysis ports and further refine venting and fluid-handling subsystems to enhance flow stability. These advancements will collectively transition the P-meter from a research prototype to a clinically deployable, intelligent, and cost-effective urine monitoring solution.

## 5. Conclusions

The P-meter effectively addresses the need for accurate, automated, and real-time UO monitoring using simple, cost-effective components. By successfully resolving prior limitations related to air venting, pump accuracy, and chamber geometry, the V3 device achieved a near-ideal performance profile, characterized by a regression slope of 0.9889, an intercept of 0.25 mL, and a minimal mean error of approximately −0.1 mL. These results confirm the device’s high accuracy and consistent agreement with reference volumes across all tested flow conditions, validating the core technical approach while identifying clear pathways for clinical translation. Thus, the V3 P-meter has been validated as a novel continuous UO monitoring system that successfully balances automation, affordability, and accuracy. The results of the V3 prototype demonstrate strong potential for clinical translation, improving patient management in critical care settings.

## Figures and Tables

**Table 1:** Validation and Performance Comparison of Prototypes V1, V2, and V3.

Prototype	R^2^	Regression Slope	Regression Intercept	Bland–Altman Mean Error	Bland–Altman Limits of Agreement (95% CI)
V1	0.99	0.9346	0.92	−1.69	94.20
V2	0.96	1.0446	1.79	3.63	96.41
V3	0.98	0.9889	0.25	−0.10	94.19

## Data Availability

Restrictions apply to the availability of some datasets generated or analyzed during this study. The raw data from animal experiments used to generate the plots and analyses presented in this article are publicly available at https://doi.org/10.5281/zenodo.18275949 (accessed on 16 January 2026). Additional design-related data, including detailed hardware designs, firmware, and related technical documentation, are not publicly available due to ongoing development and intellectual property protection activities and are intended to be disclosed as part of future patent filings. Further inquiries may be directed to the corresponding authors.

## Supplementary Materials

The following supporting information can be downloaded at: https://www.mdpi.com/article/10.3390/s26030849/s1, Table S1: Current Automated UO Monitoring Devices; Figure S1: OCB350L250Z Sensor Module; Figure S2: OCB350L250Z Sensor Output; Figure S3: Atlas Scientific EZO-PMP Pump; Figure S4: Microcontroller Arduino Mega 2560; Table S2: Hardware evolution across P-meter prototypes.

## Abbreviations

The following abbreviations are used in this manuscript:

AKI	Acute Kidney Injury
CPU	Central Processing Unit
g	Gram
I/O	Input-Output
ICU	Intensive Care Units
IDE	Integrated Development Environment
IoT	Internet Of Things
*n*	Sample Size
P-meter	Automated Urine Flow Meter
Q	Pump Flow Rate
R^2^	Coefficient Of Determination
RAM	Random-Access Memory
SD	Standard Deviation
*t*	Time
UART	Universal Asynchronous Receiver/Transmitter
UO	Urine Output
USG	Urine Specific Gravity
*V*	Volume
V1	Prototype P-meter Version 1
V2	Prototype P-meter Version 2
V3	Prototype P-meter Version 3
