# Supplementary material for: Development and Validation of an Optical Sensor-Based Automated Urine Flow Meter for Real-Time Patient Monitoring
Source: Sensors (Basel). 2026 Jan 28;26(3):849. doi: 10.3390/s26030849 (PMC12899953; doi:10.3390/s26030849)
Supplement: Supplementary file 1 [file sensors-26-00849-s001.zip › sensors-4057651-supplementary.pdf]

Supplementary Information

Development and Validation of an Optical Sensor-Based Automated Urine Flow Meter for Real-Time Patient Monitoring

Piyush Hota[1,3,4,6], Adithya Shyamal Pandian[2,3,4,6], Rodrigo E. Domínguez[3,4,7], Manni Mo[3,4,6], Bo Fu[3,4], Sandra Miranda[3,4], Pinar Cay Durgan[3,4,6], Dheeraj Sirganagar[3,6], Michael Serhan[3,4], Peter Serhan[3,4], Kevin Abi Karam[3,4,7], Naomi M. Gades[5], Peter Wiktor[3], Leslie Thomas[4,6*], Mary Laura Lind[1,3,4,6*], and Erica Forzani[1,2,3,4,6,7*]

[1] School for Engineering of Matter, Transport & Energy, Ira A. Fulton School of Engineering, Tempe, AZ; phota@asu.edu (P.H.).

[2] School of Electrical, Computer, and Energy Engineering, Ira A. Fulton School of Engineering, Tempe, AZ; ashyamal@asu.edu (A.S.P.).

[3] Center for Bioelectronics and Biosensors, Biodesign Institute, Arizona State University, Tempe, AZ 85287, 1001 S McAllister Ave., Tempe, AZ 85281, U.S.A.; redoming@asu.edu (R.E.D.); mannimo@asu.edu (M.M.); bofu4@asu.edu (B.F.); smiran14@asu.edu (S.M.); pcay1@asu.edu (P.C.D.); dsirgana@asu.edu D.S.); mserhan@asu.edu (M.S.); pserhan@asu.edu (P.S.); kabikar1@asu.edu (K.A.K.); Peter.Wiktor@asu.edu (P.W.).

[4] Medical Devices and Methods Laboratory, Health Futures Center, Arizona State University, 6161 E. Mayo Blvd., Phoenix, AZ 85054, U.S.A.

[5] Department of Comparative Medicine, Mayo Clinic Arizona, 13400 East Shea Boulevard, Scottsdale, AZ 85259, U.S.A.; gades.naomi@mayo.edu (N.M.G.).

[6] Department of Nephrology and Hypertension, Mayo Clinic Arizona, 5777 E Mayo Boulevard, Phoenix, AZ 85054, U.S.A.

[7] Phoenix Children's Research Institute, Department of Child Health, College of Medicine – Phoenix, University of Arizona, 475 N. 5th St., Phoenix, AZ 85004.

* Correspondence: eforzani@arizona.edu (E.F.); mllind@asu.edu (M.T.); thomas.leslie@mayo.edu (L.T.).

Table of Contents

Table S1. Current Automated UO Monitoring Devices.. 3

Figure S1. OCB350L250Z Sensor Module [39] 4

Figure S2. OCB350L250Z Sensor Output... 4

Figure S3. Atlas Scientific EZO-PMP Pump [40] 4

Figure S4. Microcontroller Arduino Mega 2560 [41].. 5

Table S2. Hardware evolution across P-meter prototypes 5

Table S1. Current Automated UO Monitoring Devices.

Device (Company)	Technology/Sensing Method	Key Features & Resolution	Status/Validation
kUO Plus [26] (Fize Medical)	Proprietary in-line sensing; incremental volume detection.	Microliter-level resolution; continuous flow-rate calculation; alarm notifications.	Commercial; minimizes user dependence and positioning errors.
Urinfo 2000 [27] (FlowSense/Baxter)	Custom infrared drop detector; dual-chamber system for precise droplet formation.	Measures volumes >1 mL; interfaces with ICU systems.	Commercial; validated against manual measurement using a cylinder reference.
ClarityRMS [18,32] (RenalSense)	Thermal method; measures temperature change from thermal energy pulses.	Cost-effective; accurate even at low volumes.	Commercial; validated in clinical trials (cardiac surgery patients) using weight measurement as the gold standard.
Accuryn (Potrero Medical) [31]	Active drainage (controlled low vacuum); fused sensors (pressure, acoustic/ultrasound).	Measures UO rate, intra-abdominal pressure (IAP), and core temperature in real-time.	FDA-approved; undergoing large-scale clinical studies (Accuryn Registry).
Sentinel (Serenno Medical)[20]	Proprietary in-line sensing module.	Continuous real-time measurement; alerts for early kidney dysfunction detection; 96% accuracy demonstrated in initial trials.	In development/clinical trials.
Otero Prototype [17]	Capacitive sensor in a 90 mL container; magnetic stopper for periodic drainage.	Minute-by-minute measurements; Bluetooth data transmission.	Laboratory testing only.
Art Medical [35]	Droplet counting; uses a photosensor and fast-gated camera for individual drop volume estimation.	Software calculates flow rate based on estimated volume and timing.	Patented, prototype stage.
Piyapema Prototype [34]	Drop generator; NTC thermistor sensing in a drip chamber.	Low-cost disposable sensor; Raspberry Pi control; IoT connectivity for cloud data storage.	Prototype stage.
Padilla Prototype [33]	Two infrared reflective sensors along the drain tube; measures travel time.	Measures UO volume and turbidity; uses an Arduino microcontroller.	Laboratory testing only (urinary system model).

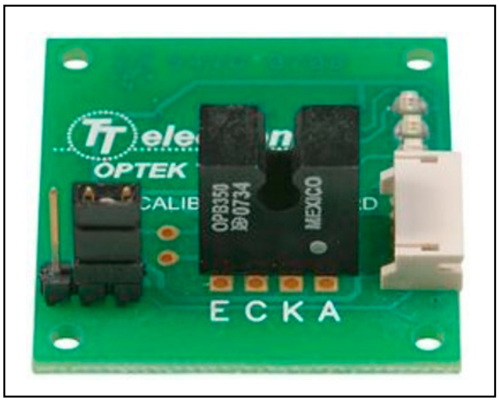

Figure S1. OCB350L250Z Sensor Module [39]

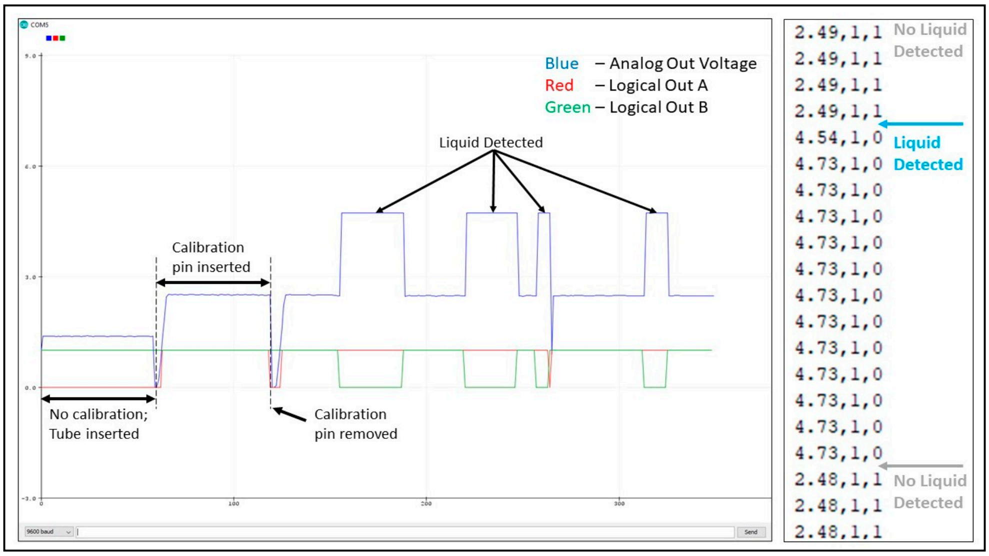

Figure S2. OCB350L250Z Sensor Output.

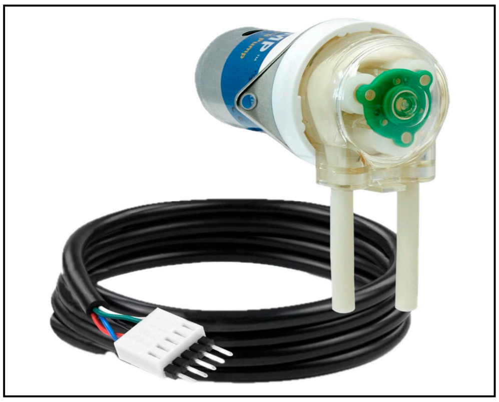

Figure S3. Atlas Scientific EZO-PMP Pump [40]

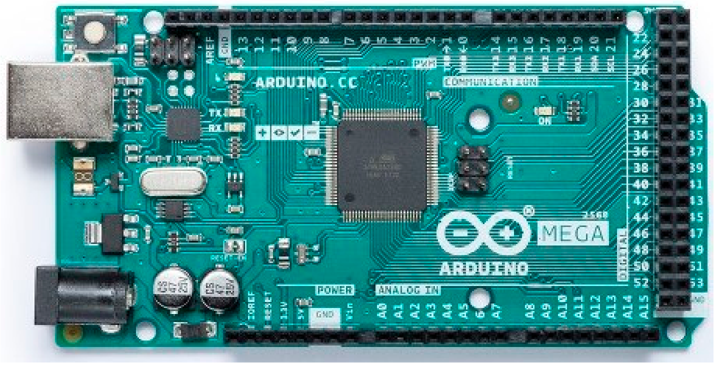

Figure S4. Microcontroller Arduino Mega 2560 [41]

Table S2. Hardware evolution across P-meter prototypes.

**Feature**	**V1**	**V2**	**V3**
Micro-controller	Arduino Mega 2560	Arduino Mega 2560	Micro-chip<br>PIC 32MX470F512H
Pump	Atlas Scientific EZO-PMP	Atlas Scientific EZO-PMP	Boxer Pumps 9QQ Stepper
Sensors	TT Electronics<br>OCB350L250Z	TT Electronics<br>OCB350L250Z	TT Electronics<br>OCB350L250Z
Chamber Volume	25ml	7.5ml	25ml (optimized)
Vent/Filter	Basic Vent	Integrated Vent	Custom Hydrophobiuc<br>PTFE membrane
Housing	Open bench	IP52 enclosure	Modular /sealed
